# Aggregation, Sedimentation, and Dissolution of Copper Oxide Nanoparticles: Influence of Low-Molecular-Weight Organic Acids from Root Exudates

**DOI:** 10.3390/nano9060841

**Published:** 2019-06-01

**Authors:** Cheng Peng, Hong Tong, Peng Yuan, Lijuan Sun, Lei Jiang, Jiyan Shi

**Affiliations:** 1 Textile Pollution Controlling Engineering Center of Ministry of Environmental Protection, College of Environmental Science and Engineering, Donghua University, Shanghai 201620, China; 2181554@mail.dhu.edu.cn (H.T.); 2181459@mail.dhu.edu.cn (P.Y.); 2 Shanghai National Engineering Research Center of Urban Water Resources Co., Ltd., Shanghai 200082, China; leilei79813@163.com; 3 Shanghai Institute of Pollution Control and Ecological Security, Shanghai 200092, China; 4 Institute of ECO-Environment and Plant Protection, Shanghai Academy of Agricultural Sciences, Shanghai 201403, China; sunliuliu2012@126.com; 5 Department of Environmental Engineering, College of Environmental and Resource Sciences, Zhejiang University, Hangzhou 310058, China

**Keywords:** root exudates, low-molecular-weight organic acids, CuO nanoparticles, aggregation, dissolution

## Abstract

The rhizosphere is an essential pathway for the uptake of metal-based nanoparticles (MNPs) by plant roots. However, the interaction between root exudates and MNPs is still unclear. In this study, we initially identified the major low-molecular-weight organic acids (LMWOAs) in the rice root exudates using hydroponics. Then, the individual LMWOAs were added to CuO nanoparticle suspensions to investigate their effects on the environmental behavior of the MNPs. The results showed that both the variety and the concentration of LMWOAs impacted the aggregation, sedimentation, and dissolution of CuO nanoparticles (NPs). Almost all LMWOAs except succinic acid inhibited the aggregation of CuO NPs by enhancing the electrostatic repulsive force between NPs. The presence of citric and oxalic acids rather than lactic acid greatly improved the stability of CuO NP suspensions, but other acids showed a low promoting and high inhibiting effect on NP sedimentation. Moreover, all the LMWOAs from root exudates facilitated the dissolution of CuO NPs with a positive dose-dependent correlation, especially formic acid. Notably, citric acid, as the most abundant LMWOAs in rice root exudates, largely determined the aggregation, sedimentation, and dissolution of CuO NPs. This study provides a better understanding on NP–plant interactions in the rhizosphere.

## 1. Introduction

Increasing applications of metal-based nanoparticles (MNPs) can eventually induce the release of MNPs into the environment during their production, transportation, use, and disposal [1]. MNPs absorbed by organisms may bind with biological macromolecules and even remain in the body producing toxic effects in the long term. Plants, as an important producer of the ecosystem, become a potential pathway for the bioaccumulation and transformation of nanoparticles in the food chain [2]. Previous studies have demonstrated that titanium dioxide nanoparticles (TiO_2_ NPs) could be translocated from soil to cucumber fruits [3], while CeO_2_ NPs in the soil could be accumulated and transferred at different trophic levels of the zucchini–cricket–wolf spider food chain [4]. Moreover, more crops will be exposed to nanoparticles than wild plants as a consequence of the wide applications of MNPs in agriculture [5,6]. In fact, the rhizosphere is the main route for plant roots to absorb environmental pollutants [7]. Therefore, understanding the environmental behavior and transformation of MNPs in the rhizospheric environment is critical to control the translocation of MNPs to the food chain and ensure the safety of agricultural products.

Generally, about 30–60% of net photosynthetic products of annual plants are distributed to the roots, and 4–70% of them are released into the rhizosphere as organic carbon [8]. Root exudates are composed of low-molecular-weight compounds such as amino acids, organic acids, sugars, and high-molecular-weight compounds such as mucus (polysaccharides) and proteins [9]. Among them, organic acids accounted for 33% of the low-molecular-weight soluble substances in the root exudates. Moreover, low-molecular-weight organic acids (LMWOAs) such as oxalic acid, citric acid, and malic acid play an important role in the chelation of heavy metals, dissolution of nutrients, and acidification in the rhizosphere. This may further impact the adsorption capacity of heavy metals by soil particles and the absorption of heavy metals by plants [10]. Previous studies showed that citric acid increased the storage of Fe and Al in *Phragmites australis* tissues around vascular bundles of rhizomes [11], and also caused a significant increase in Mn, Zn, Cu, Pb, and Cd accumulation [12,13]. However, Zhan et al. [14] found that high concentrations of citric acid reduced the Pb content in the plant shoot by destroying the structure of compounds formed by metal ions and organic acids. Additionally, Wu et al. [15] reported that oxalic, citric, and malic acids had no significant effect on the Cu content of mustard leaves, since the applied organic acids were decomposed and lost their effect. Thus, the LMWOAs from root exudates largely determine the bioavailability of metal elements and their binding species in the plant rhizosphere.

Root exudates may drive the transformation of MNPs in the rhizosphere, thus altering their bioavailability. During the uptake and translocation of MNPs, a series of MNP biotransformations occurs on the surface of plant roots or in plants. Stegemeier et al. [16] pointed out that alfalfa (*Medicago sativa* L.) root exudates partially dissolved Ag_2_S NPs and further enhanced their bioavailability to plants. Similarly, Zhang et al. [17] reported that Yb^3+^ concentration released from Yb_2_O_3_ NPs in the rhizosphere solution of cucumber was higher than that in the solution without plant, indicating that the interaction of MNPs with plant promoted the dissolution of insoluble rare element NPs. Furthermore, they found that the uptake and accumulation of CeO_2_NPs by plants, especially shoots, was mainly attributed to the dissolution of CeO_2_ NPs caused by the LMWOAs in root exudates [17]. In contrast, Huang et al. [18] indicated that the association of small organic acids with Cu NPs significantly decreased Cu uptake and bioaccumulation in plants. Hence, the information on the interaction mechanisms between root exudates and MNPs is still limited. Moreover, the key component of organic acids from root exudates that determines the environmental fate of MNPs is yet unrevealed.

Copper oxide nanoparticles (CuO NPs) are typical MNPs released into the environment and may pose potential risks to the quantity and quality of agricultural products [19]. In this study, the rice plant was used as a model plant since it is the most consumed staple food in the world. The main components of rice root exudates were identified, and the interaction between each LMWOA from the root exudates with CuO NPs was studied. The hydrodynamic size distribution and surface charge of particles, sedimentation, and dissolution of CuO NPs were investigated for clarifying the effects of various organic acid components from the root exudates on the behavior of CuO NPs. Besides, the Derjaguin–Landau–Verwey–Overbeak (DLVO) theory was used to analyze the interaction energy between CuO NPs to further reveal the underlying mechanism by which LMWOAs affected the aggregation of CuO NPs. Therefore, this study not only is beneficial to elucidate the absorption mechanism of MNPs by plants in the rhizosphere but also can provide a scientific basis for the comprehensive assessment of the environmental risks of MNPs.

## 2. Materials and Methods

### 2.1. Characterization of CuO NPs

CuO NPs were purchased from Shanghai Macklin Biochemical Co., Ltd., Shanghai, China. The product information provided by the manufacturer showed that CuO NPs had a density of 6.320 g/cm^3^, an average particle size of 40 nm, and a purity higher than 99.5%. The particle size and morphology of CuO NPs were characterized by a transmission electron microscope (TEM, H-7650, Hitachi High Technologies, Tokyo, Japan).

### 2.2. Plant Culture

Rice (*Oryza sativa* L.) seeds (Zhongzao No. 39) were provided by Zhejiang Wuwangnong Seeds Shareholding Co. Ltd. (Hangzhou, China). The method of plant culture has been described in a previous study [20]. Briefly, rice seeds were sterilized by a 10% (*v*/*v*) NaClO solution and germinated in the dark for a week. Then, the seedlings were transferred to a nutrient solution for additional two weeks. Uniform 21 day seedlings were chosen for subsequent experiments. The formula of the nutrient solution was from the International Rice Research Institute [21]. The pH of the nutrient solution was adjusted to 5.5 to maintain the normal growth of rice seedlings, and the nutrient solution was refreshed every 3 days. Plant culture was conducted in an artificial climate chamber (16 h of light and 8 h of dark, 150 μmol/(m^2^·s) of light intensity, 28 °C/24 °C of day/night temperature, 70%–75% of relative humidity).

### 2.3. Collection of Root Exudates

Fifty rice seedlings were thoroughly washed with deionized water. Then, the roots were completely submerged in a conical flask with 500 mL of sterile deionized water. Root exudates were immediately collected for 6 h. Afterwards, the root exudates were successively filtered through quantitative filter papers and filter membranes with a pore size of 0.45 μm to remove root debris and microorganisms. The filtered solution was pre-frozen and lyophilized at −40 °C and 0.280 mbar pressure in a freeze-dryer.

### 2.4. Identifications of LMWOAs in Root Exudates

The identification of LMWOAs in root exudates was performed by Shanghai Fuxin Chemical Technology Service Co., Ltd. (Shanghai, China) All organic acid standards (citric, oxalic, tartaric, formic, malic, lactic, acetic, and succinic acids) used for HPLC were chromatographic-grade and were purchased from Sigma-Aldrich (St. Louis, MO, USA). All solutions and standards were prepared with Milli-Q water (18.2 MΩ; Millipore, Bedford, MA, USA). The compounds in root exudates were analyzed using a HPLC system (U3000, Thermo Fisher Scientific Inc., Bremen, Germany). Separation was performed on a Syncronis C_18_ column (250 mm × 4.6 mm) with 5 μm particle size (Thermo Fisher Scientific Inc., Waltham, MA, USA) at a column temperature of 30 °C. The mobile phase consisted of 5% acetonitrile and 95% Milli-Q water with 0.5% phosphoric acid at the flow rate of 0.8 mL/min. The wavelength of the UV detector was set at 210 nm. Standard organic acids were also chromatographed under the same conditions. The chemical compounds were identified by comparing the retention times, major peaks, and peak areas of the standard compounds with those of the root exudate samples [22].

### 2.5. Aggregation Analysis of CuO NPs

**Figure 1:**
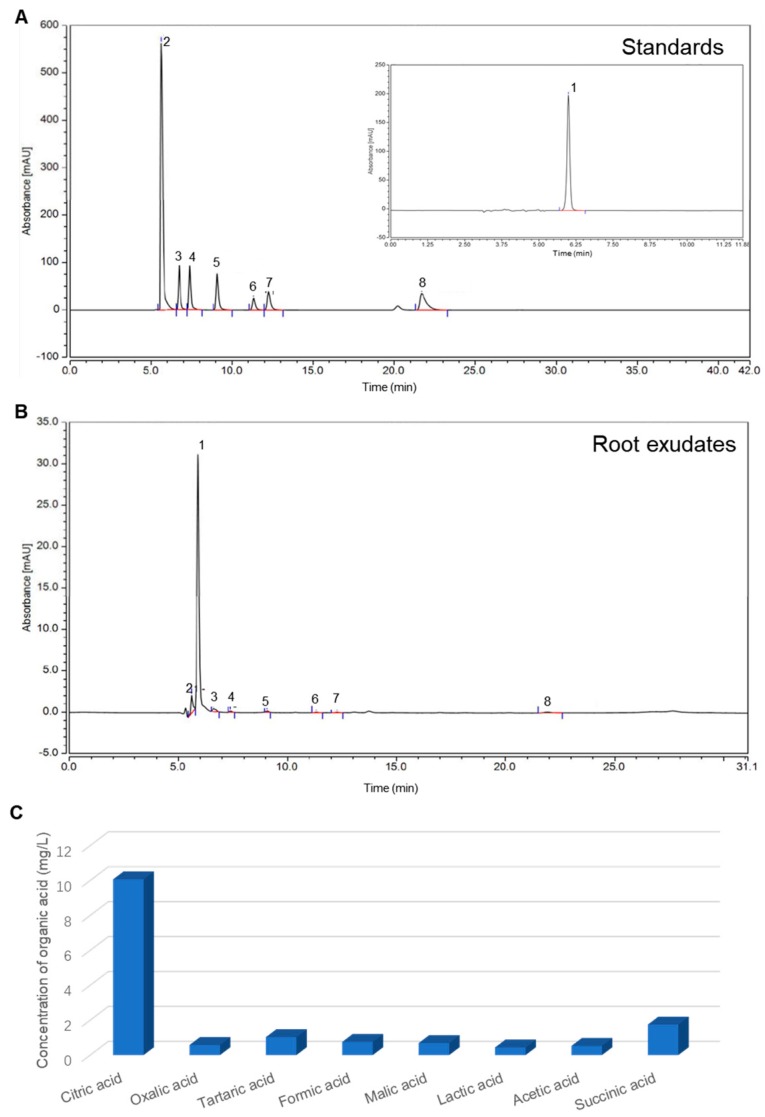
Chromatogram of organic acid standards (**A**) and root exudates (**B**) and concentrations of organic acids in the root exudates (**C**) detected by HPLC. The peaks are assigned to different organic acids: 1, citric acid; 2, oxalic acid; 3, tartaric acid; 4, formic acid; 5, malic acid; 6, lactic acid; 7, acetic acid; 8, succinic acid.

The CuO NP stock solution (1000 mg/L) was prepared by adding CuO NPs into Milli-Q water with bath sonication for 30 min at room temperature. Organic acid standards were dissolved in Milli-Q water to prepare stock solutions with an initial mass concentration of 100 mg/L. Besides, in order to investigate the effect of the LMWOA mixture on the fate of CuO NPs, we mixed eight kinds of LMWOAs with the identified contents of LMWOAs in root exudates, as shown in Figure 1. CuO NP and organic acid stock solutions were added into the bulk solution (10 mM NaCl) at a final concentration of 100 mg/L CuO NPs, with organic acid gradient concentrations (0, 1, and 10 mg/L) and an LMWOA mixture. The concentration of CuO NPs was selected by referring to previous studies on the interaction between CuO or Cu NPs and plants under hydroponic condition [18,23,24,25].

After the mixtures equilibrated for 1 h at room temperature, the size distribution and zeta potential of NPs were detected by Zetasizer Nano ZS-90 (Malvern Instruments Ltd., Malvern, UK) based on dynamic light scattering (DLS) analyses [26]. The classic DLVO theory was used to explain the aggregation behavior of CuO NPs with various LMWOAs. We calculated van der Waals (vdW) attractive energy, electrostatic interaction (electrical double layer, EDL) energy between particles, as well as total interaction energy between two interacting particles as described in a previous study [26].

### 2.6. Sedimentation Measurements of CuO NPs

The sedimentations of CuO NPs suspended in different concentrations of LMWOAs were monitored using a UV–vis spectrophotometer (UV-6100PC, Metash Instrument Co., Ltd., Shanghai, China). The absorbance of CuO suspensions was measured in a quartz cuvette at the wavelength of 240 nm over 4 h with 2 min intervals at 25 °C. The first-order kinetics model combined with the Stokes formula was used to fit the sedimentation curve [26].

### 2.7. Dissolution Detection of CuO NPs

The mixtures of CuO NPs (100 mg/L) and different organic acids were kept for 6, 12, and 24 h at room temperature (~25 °C) until the suspensions were centrifuged at 10,000 *g* for 30 min. Then, Cu^2+^ concentrations in the filtrates were detected by a flame atomic absorption spectrometer (Z-2000, Hitachi High Technologies, Tokyo, Japan). The dynamic dissolution of CuO NPs can be described by a linear empirical rate law based on zero order kinetics for solid phase [27]. The dissolution rate was calculated using the following equation:

*C* = *C*_0_ + *kt*

where *k* is the dissolution rate, and *C* and *C*_0_ are the instantaneous concentrations of Cu^2+^ in the suspensions at time *t* and 0, respectively.

### 2.8. Statistical Analysis

One-way analysis of variance (ANOVA) with least significant difference (LSD) test was used in the significance analysis (SPSS Version 19.0, SPSS Inc., Chicago, IL, USA); *p* < 0.05 was considered as a significant difference. The dissolution data in the figures are the mean ± standard deviation (SD).

## 3. Results

### 3.1. LMWOAs in Root Exudates

Root exudates mainly consist of LMWOAs, which may largely impact the environmental behavior and fate of MNPs. Here, the specific components of rice root exudates were analyzed to determine the root exudate profiles. Eight LMWOAs were detected using HPLC, including citric, oxalic, tartaric, formic, malic, lactic, acetic, and succinic acids (Figure 1A). By matching the samples’ chromatograms with those of the standards, all LMWOAs were identified in the rice root exudates (Figure 1B). Among them, the concentration of citric acid was the highest, reaching 10.07 mg/L and being 10–20 times as high as those of other LMWOAs (Figure 1C). The concentrations of succinic acid and tartaric acid were higher than 1 mg/L, while the concentrations of other LMWOAs were much lower (0.53–0.76 mg/L) in the root exudates.

### 3.2. Aggregation of CuO NPs in LMWOAs from Root Exudates

**Figure 2:**
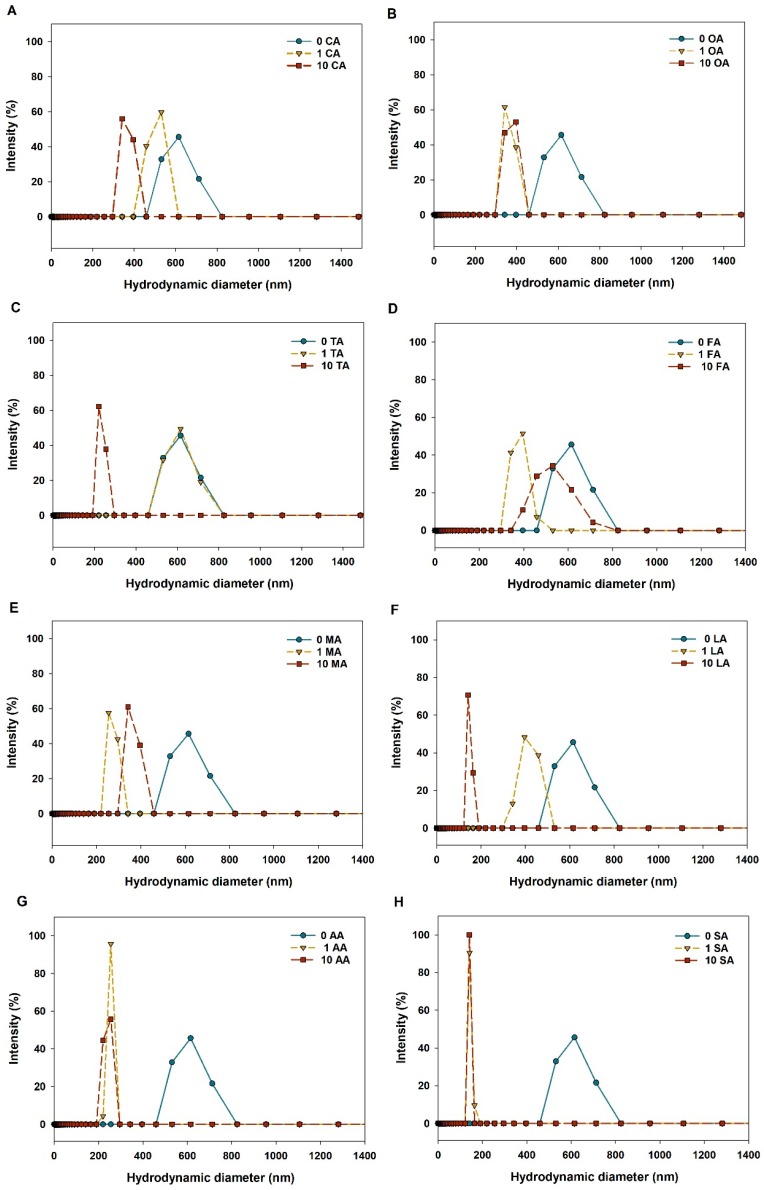
Size distribution of CuO nanoparticles (NPs) (100 mg/L) in aqueous solutions with different low-molecular-weight organic acids (LMWOAs). (**A**) CA: Citric acid; (**B**) OA: Oxalic acid; (**C**) TA: Tartaric acid; (**D**) FA: Formic acid; (**E**) MA: Malic acid; (**F**) LA: Lactic acid; (**G**) AA: Acetic acid; (**H**) SA: Succinic acid.

CuO NPs used in the study was subsphaeroidal particles with an average particle size less than 40 nm (Figure S1). Figure 2 shows that the size distribution of CuO NPs was affected by the variety and concentration of organic acids in the solution with 10 mM NaCl. At low concentration of LMWOAs (1 mg/L), all organic acids except tartaric acid reduced the hydrodynamic diameter of CuO NPs compared to the control (without organic acids), suggesting that even low level of organic acids could still improve the dispersity of CuO NPs. At a high level of organic acids (10 mg/L), the size of CuO NP aggregates was further decreased to approximately 200 nm by citric, tartaric, and lactic acids. Especially, succinic acid and high concentrations of lactic acid greatly enhanced the dispersity of CuO NP suspensions with respect to the control. In contrast, no dose-dependent effect of LMWOA concentrations on NP aggregation could be found for solutions of oxalic, formic, and malic acids.

**Figure 3:**
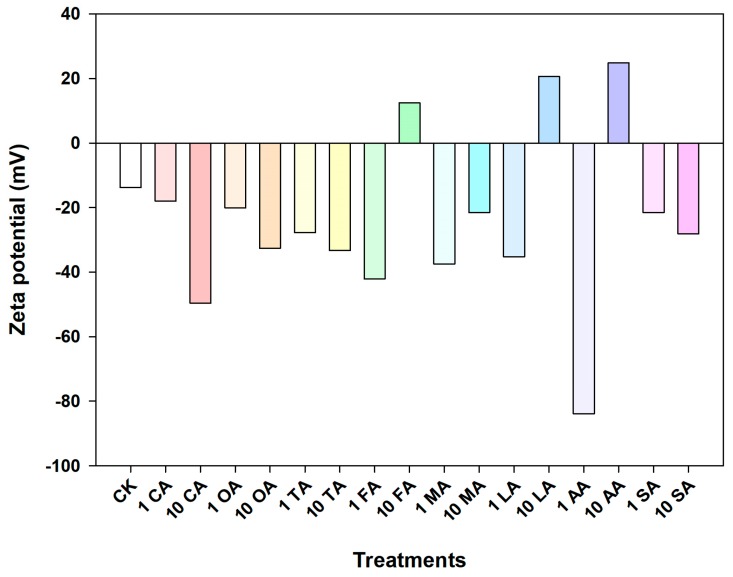
Zeta potential of CuO NPs (100 mg/L) in aqueous solutions with different LMWOAs.

The zeta potential of CuO NPs was detected over a range of LMWOA concentrations as shown in Figure 3. The surface charges of CuO NPs were determined by both the properties of LMWOAs and their concentrations. A negative concentration-dependent effect of citric, oxalic, tartaric, and succinic acids was found on the zeta potential of CuO NPs. Meanwhile, the zeta potential of CuO NPs was reduced from −13.8 mV (control) to even −83.9 mV with low concentrations of formic, lactic, and acetic acids; however, the surface charges of CuO NPs were switched from negative to positive in the presence of increasing concentrations of those three organic acids.

**Figure 4:**
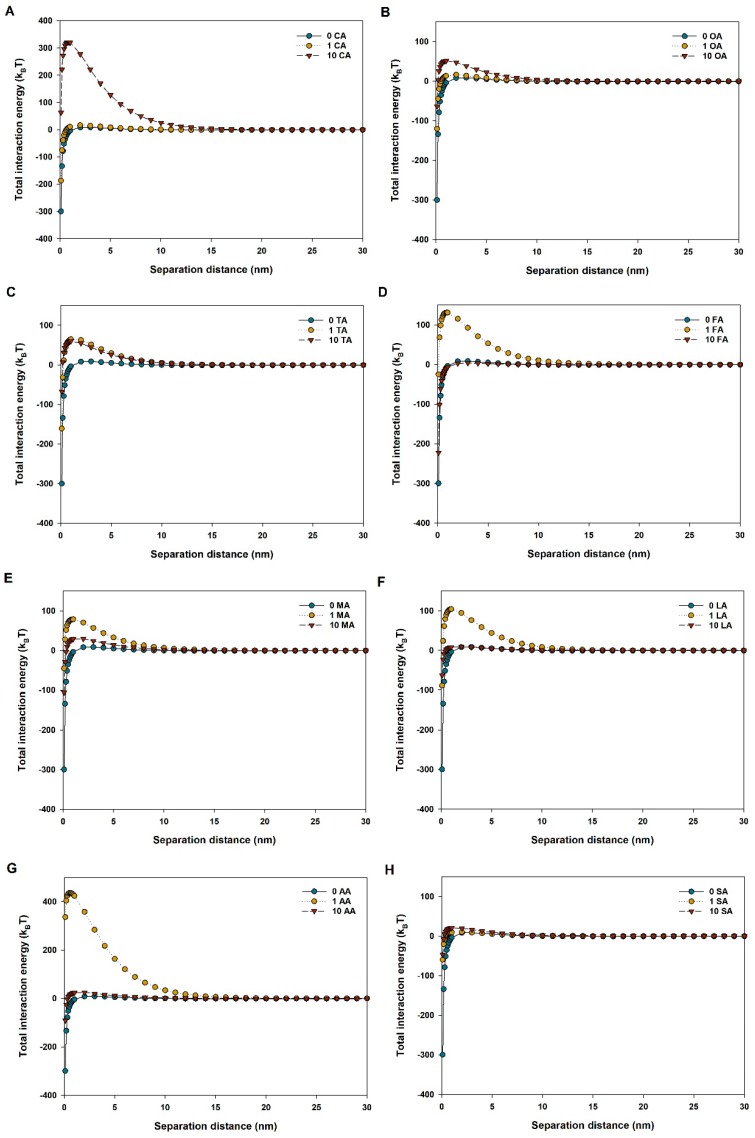
Calculated Derjaguin–Landau–Verwey–Overbeak (DLVO) total interaction energy between two CuO NPs (100 mg/L) in the presence of different LMWOAs. (**A**) CA: Citric acid; (**B**) OA: Oxalic acid; (**C**) TA: Tartaric acid; (**D**) FA: Formic acid; (**E**) MA: Malic acid; (**F**) LA: Lactic acid; (**G**) AA: Acetic acid; (**H**) SA: Succinic acid.

As calculated by the DLVO theory, the vdW force between CuO NPs was slightly increased in the presence of LMWOAs (Figure S2). Also, we found a negative relationship between increasing concentrations of LMWOAs and vdW force between CuO NPs. However, the electrostatic repulsive force of CuO NPs was affected not only by the variety of LMWOAs but also by LMWOA concentration. The presence of most LMWOAs enhanced the electrostatic repulsive force between NPs with respect to the control. This was especially evident with 100 mg/L of citric acid and oxalic acid (Figure S3A–C,E,G). Moreover, low concentrations of tartaric, malic, and acetic acids further increased the repulsive EDL energy compared to high concentrations of the same acids (Figure S3C,E,G). The repulsive EDL energy of CuO NPs was significantly increased to about 150 k_B_T by low concentrations of formic acid and lactic acid, but was reduced to 18 k_B_T by high levels of the two LMWOAs, which was even lower than that of the control (Figure S3D,F). Additionally, succinic acid showed no or even a negative impact on the electrostatic repulsive force between NPs (Figure S3H). Thus, the total interaction force between NPs was repulsive in various LMWOA solutions, as shown in Figure 4. Particularly, a big energy barrier up to 300–400 k_B_T between CuO particles could be found with the exposure to high citric acid and low acetic acid concentrations (Figure 4A,G). Yet, the net interaction force between NPs was closed to zero in the presence of succinic acid (Figure 4H).

### 3.3. Sedimentation of CuO NPs in LMWOAs from Root Exudates

**Figure 5:**
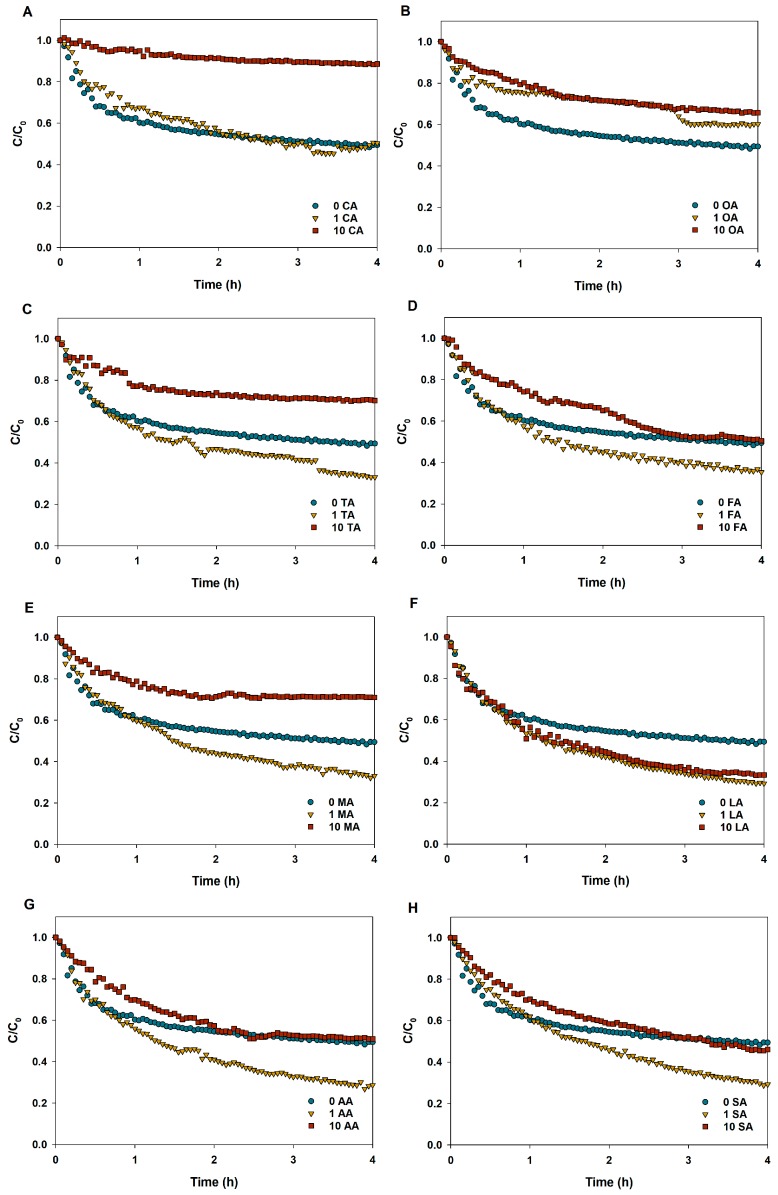
Sedimentation rate of CuO NPs (100 mg/L) in aqueous solutions with different LMWOAs. (**A**) CA: Citric acid; (**B**) OA: Oxalic acid; (**C**) TA: Tartaric acid; (**D**) FA: Formic acid; (**E**) MA: Malic acid; (**F**) LA: Lactic acid; (**G**) AA: Acetic acid; (**H**) SA: Succinic acid.

Figure 5 shows the sedimentation characteristics of CuO NPs in different LMWOA solutions with varying concentrations. Table 1 presents the sedimentation rates of CuO NPs with different LMWOA contents in aqueous solution via fitting the first-order kinetics model. The low concentration of citric acid had a slight effect on CuO NP sedimentation. However, the sedimentation rate of CuO NP suspensions was drastically reduced from 0.121 h^−1^ in the control to 0.028 h^−1^ in the 100 mg/L citric acid solution (Figure 5A and Table 1), suggesting that the presence of a high concentration of citric acid greatly improved the stability of CuO NP suspensions. The addition of oxalic acid also inhibited the sedimentation of CuO aggregates but without a dose-dependent correlation (Figure 5B). Moreover, low concentrations of tartaric, formic, malic, acetic, and succinic acids obviously accelerated the sedimentation of CuO NPs, whereas high concentrations of the five acids showed the opposite effect (Figure 5C–E,G–H). Notably, the positive impact of high organic acids (formic, acetic, and succinic acids) on the stability of CuO NP suspensions could only last for 2 h. Additionally, both low and high concentrations of lactic acid facilitated the sedimentation of CuO NPs (Figure 5F).

### 3.4. Dynamic Dissolution of CuO NPs in LMWOAs from Root Exudates

**Figure 6:**
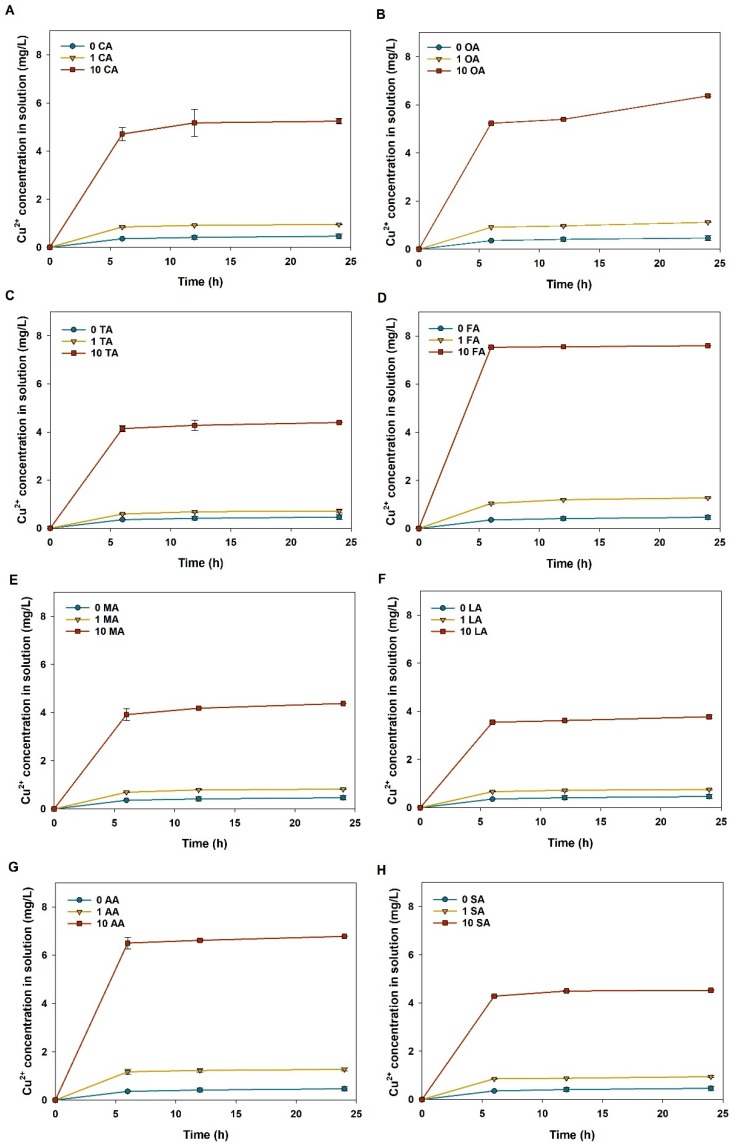
Dissolution of CuO NPs (100 mg/L) in aqueous solutions containing different LMWOAs. The values of Cu concentration are given as mean ± SD of triplicate samples. (**A**) CA: Citric acid; (**B**) OA: Oxalic acid; (**C**) TA: Tartaric acid; (**D**) FA: Formic acid; (**E**) MA: Malic acid; (**F**) LA: Lactic acid; (**G**) AA: Acetic acid; (**H**) SA: Succinic acid.

Apparently, all the LMWOAs from the root exudates promoted the dissolution of CuO NPs with a positive dose-dependent correlation (Figure 6). The dissolution of CuO NPs almost reached the dissolution equilibrium within 6 h, except for the solution containing 10 mg/L oxalic acid (Figure 6). CuO NP suspensions (100 mg/L) released 0.36 mg/L Cu^2+^ in the control solution during 6 h, while dissolved 0.60–1.17 mg/L Cu^2+^ in the presence of low concentrations of LMWOAs (1 mg/L) and even more than 4 mg/L Cu^2+^ in the presence of high concentrations of LMWOAs (10 mg/L). In addition, another rising trend of CuO NP dissolution could be found from 12 to 24 h in the presence of high concentrations of oxalic acid (Figure 6B). At steady state, Cu^2+^ concentration was slightly enhanced from 0.46 mg/L in the control to 0.72–0.82 mg/L in the solutions containing low concentrations of tartaric, malic, and lactic acids; also, 1 mg/L of formic acid and acetic acid drastically increased the dissolution of CuO NPs to more than 1.26 mg/L Cu^2+^. Furthermore, the highest dissolution from CuO NP suspensions corresponded to 7.59 mg/L in 10 mg/L formic acid solution at 24 h, which was 16 times higher than that measured in the control (Figure 6D). Thus, high level of formic acid showed a strong positive effect on the dissolution of CuO NPs. Also, all LMWOAs from the root exudates enhanced the dissolution rate of CuO NPs in aqueous solution (Table 2). For instance, the dissolution rate of CuO NPs was increased from 0.06 mg/(L·h) in the control to 0.141 and 0.785 mg/(L·h) in the presence of 1 and 100 mg/L citric acid, respectively. When considering the low levels of LMWOAs, lactic acid greatly promoted the dissolution rate of CuO NPs to 0.111 mg/(L·h), while, when considering the high levels of LMWOAs, formic acid dramatically elevated the NP dissolution rate to 1.256 mg/(L·h). Yet, both high and low concentrations of lactic acid only slightly improved the dissolution rate of CuO NPs to 0.111–0.591 mg/(L·h), suggesting that lactic acid had a weaker effect on the dissolution of CuO NPs compared to other LMWOAs.

### 3.5. Effect of an LMWOA Mixture on the Fate of CuO NPs

**Figure 7:**
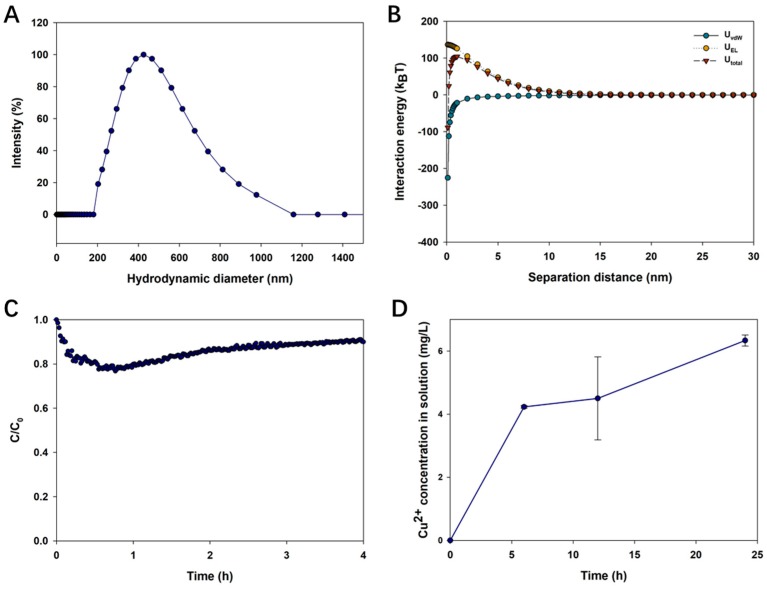
Size distribution (**A**); interaction energy (**B**); dynamic sedimentation (**C**); and dynamic dissolution (**D**) of CuO NPs in the LMWOA mixture.

Figure 7 shows the impact of an LMWOA mixture on the environmental behavior of CuO NPs. The results demonstrated that the average hydrodynamic diameter and zeta potential of CuO NPs in solution with an LMWOA mixture were 415.10 ± 10.60 nm and −35.23 ± 2.81 mV, respectively (Figure 7A). Moreover, the presence of the LMWOA mixture reduced the vdW force but enhanced the EDL energy of CuO NPs, thus inducing the increase of total interaction energy between CuO NPs to 104 kBT (Figure 7B). In Figure 7C, the stability of CuO NPs in solution declined in the first hour but then increased and remained stable during the last 3 h. The dissolution of CuO NPs was also dramatically enhanced to 4.23 mg/L at 6 h and further increased to 6.33 mg/L at 24 h with the addition of the LMWOA mixture (Figure 7D). Yet, the dissolution of CuO NPs in the LMWOA mixture did not reach a solubility equilibrium in 24 h. The results suggested that the LMWOA mixture inhibited the aggregation and sedimentation of CuO NPs and promoted the dissolution of CuO NPs.

## 4. Discussion

LMWOAs are the most abundant and reactive components of root exudates. The interaction of MNPs with LMWOAs is critical for assessing NPs environmental behavior in the rhizosphere. Organic acids from root exudates can directly impact the species of heavy metals in soil solution via acidification, precipitation, chelation, and redox reactions, thus affecting the uptake of heavy metals by plants [28]. A previous study demonstrated that the binding of Cu NPs with root exudates significantly reduced the uptake and accumulation of Cu in plants [18]. Actually, the impact of LMWOAs on the fate of MNPs was largely determined by the variety and content of LMWOAs. This present study identified eight LMWOAs from rice root exudates, in which citric acid appeared to be a major constituent (Figure 1). The specific components of LMWOAs were basically consistent with those identified in previous studies, but their concentrations were different [29,30], as a result of different cultivars, growth media, and growth stage of the rice plants [31]. In fact, these LMWOAs are also ubiquitous in root exudates derived from other plant roots besides rice. For instance, Zhao et al. [32], using GC–MS, demonstrated that citric, succinic, and malic acids were the most abundant LMWOAs in cucumber root exudates; these organic acids play an important role in the interaction between plants and Cu NPs.

The nanoparticle aggregation state is a vital factor which not only can alter the NP distribution in the environment, but also can impact the migration characteristics and biological interactions of NPs. The presence of all LMWOAs decreased the hydrodynamic diameter of CuO NPs, thus alleviating the aggregation of CuO NPs (Figure 2). The result is consistent with those of previous studies [26,33]. Actually, the aggregation state of NPs was determined by the interaction between LMWOA type and concentration, and the organic acid concentration might be the most significant factor [34]. However, Pettibone et al. [35] found that oxalic acid destabilized TiO_2_ NP suspensions as a result of the adsorption of oxalate on the NP surface. The different influence of oxalic acid on NP aggregation may result from different initial surface charges between CuO NPs and TiO_2_ NPs. Figure 3 shows that the zeta potential of CuO NPs was −13.8 mV in solution without LMWOAs, which is close to the value reported in our previous study [26]. The zero point charge pH (pHzpc) of CuO NPs was pH 6.21 in the absence of organic matter [26], indicating that the surface charge of CuO NPs is mostly positive when the pH is lower than 6.21 and turns to negative when the pH is higher than 6.21. Yet, the addition of organic acid can alter the isoelectric point of NPs [34]. Citric, oxalic, and tartaric acids decreased the zeta potentials of CuO NP aggregates with increasing concentrations of LMWOAs (Figure 3). Furthermore, more negative surface charges on NPs led to the enhancement of the electrostatic repulsive force between NPs (Figure 4A–C), thus relieving NP aggregation. Also, a recent study found that both citric acid and tartaric acid decreased the zeta potential of Ag NPs in water [36]. Even 1 mg/L of formic, malic, lactic, and acetic acids showed pronounced inhibiting effects on CuO NP aggregation (Figure 2D,E). This can be attributed to the lower zeta potential of CuO NPs at low levels of these LMWOAs, which further increased the total interaction energy barrier between two CuO NPs by magnifying the electrostatic repulsion of NPs [34]. Besides, even if the negative charges on CuO NP surfaces were shifted to positive charges by high concentrations of formic, lactic, and acetic acids, these LMWOAs did not promote the aggregation of CuO NPs, which may be explained by the fact that NP dissolution can also decrease the size of NP aggregate and further alter the aggregation state of NPs. Notably, the addition of succinic acid slightly elevated the negative surface charges of CuO NPs but reduced the electrostatic repulsion energy between NPs (Figure 3 and Figure S3H). The steric effect may play a key role in the interaction between succinic acid and CuO NPs, thus hindering NP aggregation. Furthermore, high levels of LMWOAs generally presented a stronger inhibition of NP aggregation (Figure 2). As shown in Figure S4, the initial pH values of CuO NP suspensions were 4.0–5.5 in the presence of 10 mg/L LMWOAs, then constantly increased to approximate 5.5 with high levels of LMWOAs in 24 h as a result of the consumption of H^+^ in the acidic solution due to the interaction with CuO NPs [37]. On one hand, the concentration of organic acids considerably impacted the zeta potential of CuO NPs by changing the suspension pH without a buffer [35]. On the other hand, the solution pH and the variable charge of surface hydroxyl groups largely determined the surface properties of NPs and their aggregation state [38]. Thus, the initial lower pH values of the solutions induced by higher organic acid contents may partly explain the smaller hydraulic diameter of CuO NPs exposed to high LMWOAs. Notably, in comparison with the LMWOA mixture, the presence of only citric acid showed a stronger positive impact on the dispersity of CuO NPs (Figure 2 and Figure 7). This may be caused by the reduced negative surface charges of CuO NPs and the corresponding electrostatic repulsive force between particles in a complex organic acid solution. The sedimentation of CuO NP dispersions was investigated to assess how LMWOAs from rice root exudates may influence the transport of MNPs into deeper layers in aquatic or soil environments. The results showed that the sedimentation rates of CuO NPs were impacted by different LMWOAs and their contents in aqueous solution (Table 2). Both citric acid and oxalic acid obviously alleviated the sedimentation of CuO NPs (Figure 5A–C,E), which is consistent with the fact that these organic acids reduced CuO NP aggregation, as mentioned previously. The sedimentation of CuO NPs occurred when the size of NP aggregates increased sufficiently and the gravitational forces on the NP aggregates were greater than the buoyancy forces [35]. This means that NP aggregates with smaller hydraulic diameters in solution with LMWOAs have smaller gravitational forces, which is beneficial to the stability of NPs. Nonetheless, Pettibone et al. [35] pointed out that there was little or no effect of oxalic acid on the sedimentation rate of TiO_2_ NPs at pH 6.5, because NP sedimentation is closely related to the NP aggregation state. However, some LMWOAs exhibited the opposite effects on the sedimentation of CuO NPs in the presence of different organic acid concentrations (Figure 5). NPs in aqueous environment can be deposited via co-settling with LMWOAs as hetero-agglomerates under gravity [39,40]. Not only aggregate size but also particle density in solution contributes to the sedimentation of NPs. Hence, the interaction between CuO NPs and diverse concentrations of LMWOAs may produce NP aggregates with different densities, thereby showing diverse influences on NP sedimentation. Additionally, some LMWOAs with high concentrations relieved NP sedimentation only for 2 h (Figure 5). The reason can be that the movement of NP aggregates caused by NP sedimentation may gradually decrease the collisions among aggregates suspended in solution, thereby affecting their consequent settling velocity [41]. In addition, the enhancement of NP stability by the LMWOA mixture (Figure 7) may have been caused by the formation of Cu compound in the LMWOA mixture via chelation.

Dissolution plays a critical role in the fate of MNPs in the environment. Especially, organic acids in the root exudates can promote the oxidative dissolution of MNPs, further enhancing the phytotoxicity of NPs [42]. The result showed that the presence of all LMWOAs significantly enhanced the extent of CuO dissolution (Figure 6 and Figure 7), which resulted from the combination of LMWOAs and NPs. CuO NPs can react with H^+^ from organic acids in a solution and release a number of Cu^2+^ ions under acid conditions [37]; meanwhile, CuO NPs or dissolved Cu^2+^ ions can combine with carboxyl groups associated with LMWOAs via complexation [18]. For instance, CuO can be dissolved by citric acid according to the following chemical equation: 3CuO + 2C_6_H_8_O_7_→Cu_3_(C_6_H_5_O_7_)_2_ + 3H_2_O [43]. Also, Zhao et al. [32] reported that citric acid in cucumber plants promoted the dissolution of Cu NPs. Moreover, the dissolution kinetics and equilibrium of CuO NPs depended on the type and concentration of LMWOAs in solution (Table 2 and Figure 6). As discussed before, the suspension pH can be lowered by increasing organic acid concentrations, which further enhanced NP dissolution [35]. On the basis of the ionization constant, the acidity of LMWOAs in rice root exudates follows the sequence: oxalic acid > tartaric acid > citric acid > malic acid > formic acid > lactic acid > succinic acid > acetic acid. However, the results demonstrated that formic acid most greatly facilitated NP dissolution (Figure 6D) because it released more H^+^ than other LMWOAs due to its relatively small molecular weight. In addition, oxalic acid had a similar positive effect on the dissolution of CuO NPs compared to citric acid (Figure 6A,B), but showed further promotion on NP dissolution at 48 h. However, Mudunkotuwa et al. [44] pointed out that oxalic acid showed a less significant effect on promoting the dissolution of aging Cu NPs than citric acid, and Huang et al. [18] demonstrated that citric and malic acids induced a greater dissolution of Cu NPs than oxalic and succinic acids due to a higher release of H^+^. This may be caused by the relative concentrations of LMWOAs and the different reaction times of organic acids with CuO NPs. In addition, tartaric acid showed a similar impact to citric acid on the dissolution of CuO NPs, since they have similar chelating abilities to Cu(II) [45]. Yet, the LMWOA mixture induced a higher dissolution of CuO NPs than citric acid alone (Figure 7), which may result from the higher chelating ability of mixed LMWOAs.

Organic acids in root exudates or plants can facilitate the uptake of nutrient elements or prevent that of toxic metals [32]. Carboxylic acids detected in the rice root exudates, such as citric, oxalic, malic, and succinic acids, are potential ligands for heavy metals. These LMWOAs can form stable extracellular complexes with various dissolved metals including Cu^2+^ and play a vital role in detoxification and tolerance of plants to heavy metals [32]. The results suggested that the aggregation, sedimentation, and dissolution of CuO NPs in the rhizosphere were largely determined by citric acid, since it is the most abundant LMWOAs in rice root exudates. However, the type and concentration of LMWOAs secreted by the roots may be changed in the natural soil–plant system. Moreover, LMWOAs in plants can be upregulated or downregulated by the exposure to MNPs [32]. Thus, further study on the interaction between root exudates and NPs in real complex soils needs to be conducted to explore the environmental behavior and fate of MNPs in the rhizosphere environment and understand how to control the uptake of MNPs by crops through roots.

## Figures and Tables

**Table 1:** Dynamic fitting of CuO NP sedimentation with different LMWOAs contents in aqueous solution.

LMWOAs	Concentration (mg/L)	Sedimentation Rate (h^−1^)	R^2^
Control	0	0.121	0.7669
Citric acid	1	0.164	0.8888
	10	0.028	0.8755
Oxalic acid	1	0.098	0.9076
	10	0.088	0.8932
Tartaric acid	1	0.223	0.9060
	10	0.066	0.7624
Formic acid	1	0.218	0.8802
	10	0.162	0.9537
Malic acid	1	0.245	0.9313
	10	0.061	0.6581
Lactic acid	1	0.268	0.9349
	10	0.238	0.9222
Acetic acid	1	0.284	0.9447
	10	0.155	0.8610
Succinic acid	1	0.286	0.9754
	10	0.176	0.9536

**Table 2:** Dynamic fitting of CuO NP dissolution with different LMWOAs contents in aqueous solution.

LMWOAs	Concentration (mg/L)	Dissolution Rate (Cu^2+^ mg·L^−1^·h^−1^)
Control	0	0.060
Citric acid	1	0.141
	10	0.785
Oxalic acid	1	0.152
	10	0.873
Tartaric acid	1	0.099
	10	0.691
Formic acid	1	0.172
	10	1.256
Malic acid	1	0.115
	10	0.652
Lactic acid	1	0.111
	10	0.591
Acetic acid	1	0.195
	10	1.085
Succinic acid	1	0.143
	10	0.714

## Supplementary Materials

The following are available online at https://www.mdpi.com/2079-4991/9/6/841/s1. The TEM image of CuO NPs, calculated vdW interaction energy and electrostatic interaction energy between two CuO NPs, and solution pH in the presence of varying LMWOAs are presented as supplementary information.
