# Supplementary material for: Aggregation, Sedimentation, and Dissolution of Copper Oxide Nanoparticles: Influence of Low-Molecular-Weight Organic Acids from Root Exudates
Source: Nanomaterials (Basel). 2019 Jun 1;9(6):841. doi: 10.3390/nano9060841 (PMC6630225; doi:10.3390/nano9060841)
Supplement: Supplementary file 1 [file nanomaterials-09-00841-s001.pdf]

*Supplementary Materials*

# Aggregation, Sedimentation, and Dissolution of Copper Oxide Nanoparticles: Influence of Low-Molecular-Weight Organic Acids from Root Exudates

**Cheng Peng[1,2,3]*, Hong Tong[1], Peng Yuan[1], Lijuan Sun[4], Lei Jiang[2], Jiyan Shi[5]***

[1] Textile Pollution Controlling Engineering Center of Ministry of Environmental Protection, College of Environmental Science and Engineering, Donghua University, Shanghai 201620, China; 2181554@mail.dhu.edu.cn (H.T.); 2181459@mail.dhu.edu.cn (P.Y.)

[2] Shanghai National Engineering Research Center of Urban Water Resources Co., Ltd., Shanghai, 200082, China; leilei79813@163.com (L.J.)

[3] Shanghai Institute of Pollution Control and Ecological Security, Shanghai 200092, China

[4] Institute of ECO-Environment and Plant Protection, Shanghai Academy of Agricultural Sciences, Shanghai, 201403, China; sunliuliu2012@126.com (L.S.)

[5] Department of Environmental Engineering, College of Environmental and Resource Sciences, Zhejiang University, Hangzhou 310058, China

* Correspondence: Cheng Peng. Email: cpeng@dhu.edu.cn; phone: (+86)-21-6779-8739.
Jiyan Shi. Email: shijiyan@zju.edu.cn; phone: (+86)-571-8898-2019.

**Number of pages:** 6

**Number of Figures:** 4

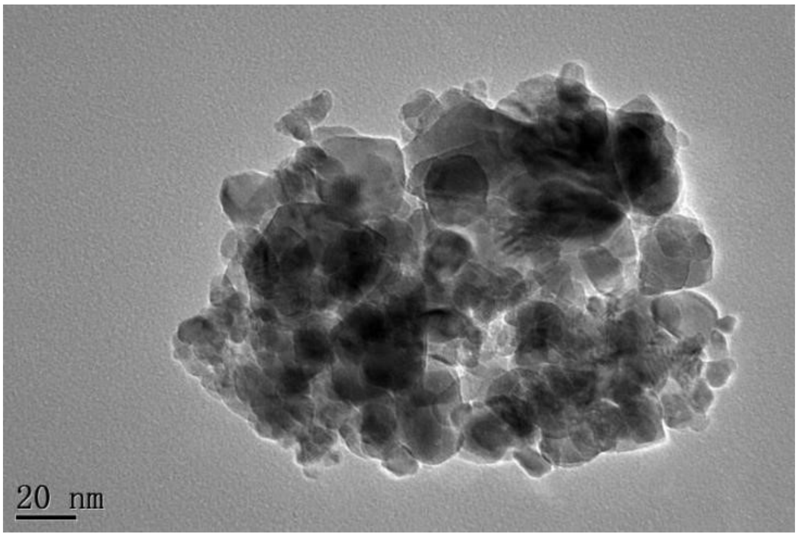


**Figure S1.** TEM image of CuO NPs

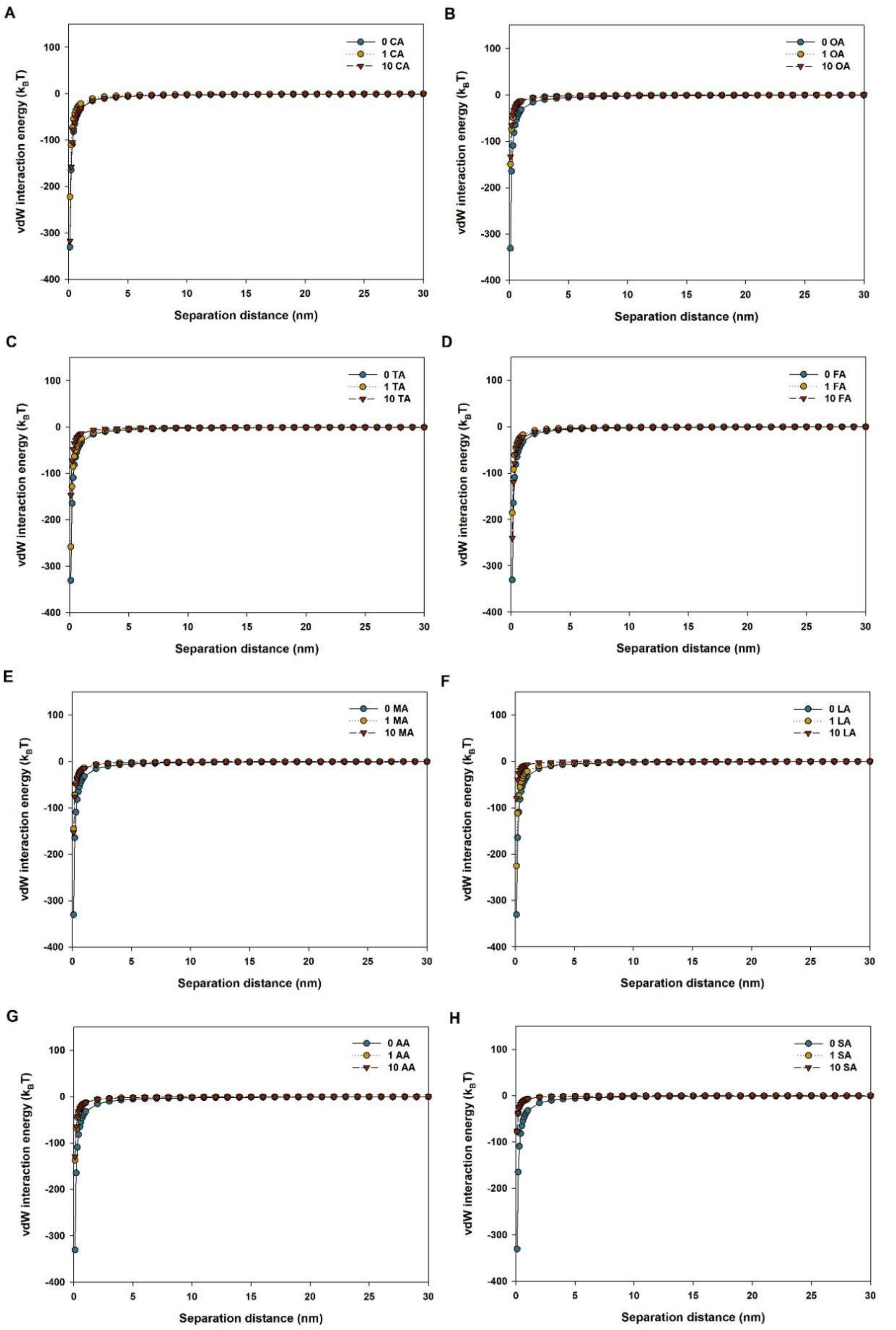


**Figure S2.** Calculated vdW interaction energy between two CuO NPs under varying LMWOAs. (**A**) CA: Citric acid; (**B**) OA: Oxalic acid; (**C**) TA: Tartaric acid; (**D**) FA: Formic acid; (**E**) MA: Malic acid; (**F**) LA: Lactic acid; (**G**) AA: Acetic acid; (**H**) SA: Succinic acid.

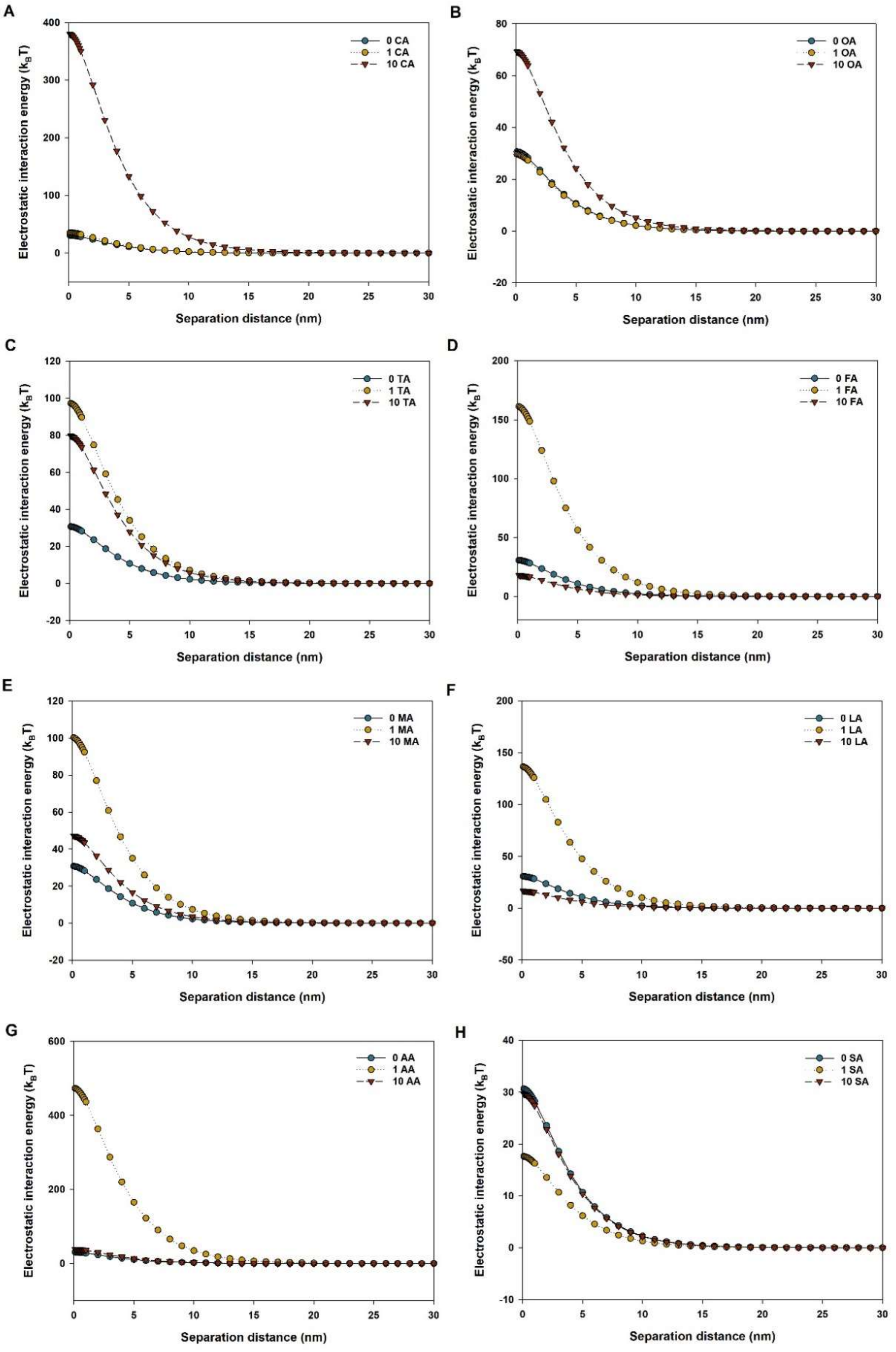


**Figure S3.** Calculated electrostatic interaction energy between two CuO NPs under varying LMWOAs. (**A**) CA: Citric acid; (**B**) OA: Oxalic acid; (**C**) TA: Tartaric acid; (**D**) FA: Formic acid; (**E**) MA: Malic acid; (**F**) LA: Lactic acid; (**G**) AA: Acetic acid; (**H**) SA: Succinic acid.

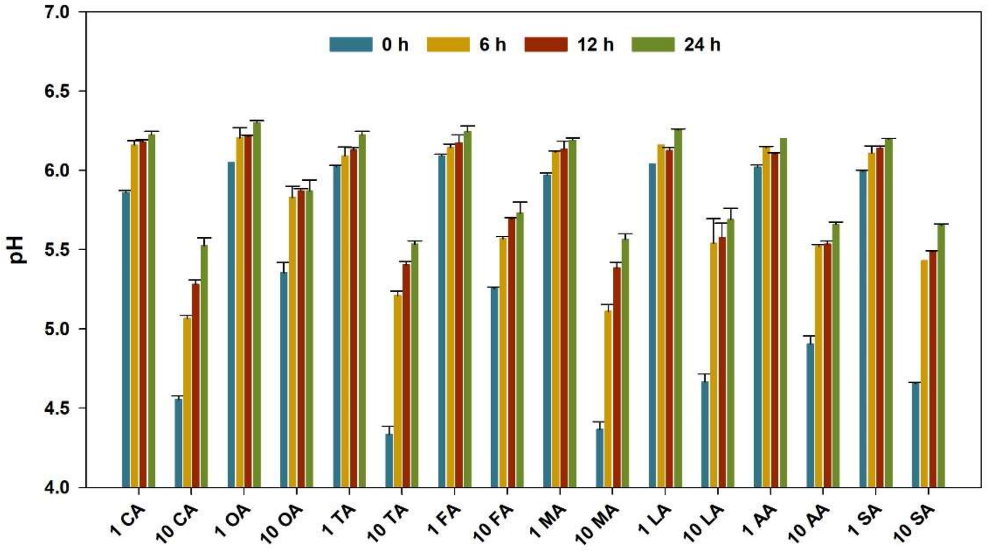


**Figure S4.** The dynamic pH of CuO NP suspension with varying LMWOAs. CA: Citric acid; OA: Oxalic acid; TA: Tartaric acid; FA: Formic acid; MA: Malic acid; LA: Lactic acid; AA: Acetic acid; SA: Succinic acid.
